# The Diversity Distribution and Climatic Niche of Samara Species in China

**DOI:** 10.3389/fpls.2022.895720

**Published:** 2022-06-17

**Authors:** Yanjun Du, Yuan Zhao, Shupeng Dong, Guoke Chen, Xinyang Wang, Keping Ma

**Affiliations:** ^1^Key Laboratory of Genetics and Germplasm Innovation of Tropical Special Forest Trees and Ornamental Plants (Ministry of Education), College of Forestry, Hainan University, Haikou, China; ^2^College of Urban and Environmental Sciences, Laboratory for Earth Surface Processes of the Ministry of Education, Peking University, Beijing, China; ^3^Dongguan Botanical Garden, Dongguan, China; ^4^State Key Laboratory of Vegetation and Environmental Change, Institute of Botany, Chinese Academy of Sciences, Beijing, China

**Keywords:** functional traits, fruit type, species diversity, distribution, climate variability, dispersal, species richness

## Abstract

Studying the distribution of samara species is of ecological and economic significance. This information helps us with understanding species dispersal mechanisms, evaluating the risk of invasive species, and the management of ecological forests. However, limited research has explored, on a large scale, the geographic distribution of samara species and their influential abiotic factors. Here, we use the distribution data of 835 vascular samara species and growth form data to explore their geographic patterns in China and the environmental determinants. We divided China into 984 grid cells and examined the relationship between the proportion of samara species and climate variables using both ordinary and spatial linear regressions for each grid cell. Total samara species richness is higher in southern China in low altitude regions and the proportion of woody samara species is significantly higher than that of herbaceous samara species. The proportion of woody samara species is higher in the northeast regions where precipitation is sufficient, winters are dry and mild, and temperature seasonality and land surface relief degree values are high. Annual precipitation and temperature seasonality are the most important climatic drivers for the distribution of woody samara species. In contrast, herbaceous samara species prefer to distribute to the areas where climate is warm and dry but have higher temperature seasonality. Temperature related variables (mean annual temperature, mean diurnal range, and temperature seasonality) are the most important drivers for the distribution of herbaceous samara species. Samara species can better adapt to climatic regions with large temperature fluctuations and dry winters. The present distribution patterns of samara species are formed by the combined adaptation of fruit traits and growth form to climate. This work contributes to predictions of the global distribution of samara species under future climate change scenarios and conservation and management for the samara species.

## Introduction

Plant functional traits allow plants to respond to changes in their living environment and/or to change in their ecosystems. Examples include leaf size, leaf lifespan, seed size, seed dispersal modes, and canopy height (Diaz and Cabido, 2001). Understanding the distribution of functional traits on a large scale can reveal the close relationships between organisms and their biotic and abiotic environments (Hampe, 2003). Fruit types influence seed dispersal (Chen et al., 2019), seed physical defense (Chen and Moles, 2018), and seedling regeneration (Chen and Giladi, 2020). Therefore, fruit type plays an important role in shaping the large-scale structure and dynamics of plant populations and communities (Levin et al., 2003; Svenning and Skov, 2007). However, large-scale distribution patterns of fruit type and the important environmental drivers affecting them remain poorly understood.

A samara is a dry, indehiscent fruit (or achene) containing one seed that is encompassed by a wing. Samaras have 25 orders, 45 families, and 140 genera of angiosperms (der Weduwen and Ruxton, 2019). Samaras are adapted for wind dispersal and grow in every habitat, including deserts, rainforests, temperate, and alpine regions, as well as on every continent except Antarctica (der Weduwen and Ruxton, 2019). On average, 10%–30% of the seed-plant flora in any one community relies on the wind to disperse seeds away from the maternal parent’s zone of influence. In some temperate plant communities, this value reaches as high as 70% (Willson et al., 1990). Thus, samaras are ecologically vital to population development due to their dispersal function. Also, several economically important genera including *Fraxinus* L. and *Pinus* L., as well as highly invasive species like *Ailanthus altissima*, *Acer ginalla* Maxim use samaras for dispersal, so studying the distribution of samara species will aid conservation planning and invasive species risk management (der Weduwen and Ruxton, 2019). Yet, the geographic distribution of samara species and their climate drivers remain unclear. Additionally, it is unclear whether the geographic distribution of the samara species and their environmental drivers differ among their various growth forms (i.e., herbaceous and woody species).

Much effort has been directed toward understanding the dynamics of samara flight (i.e., first dispersal process) as it is easily observable and measurable, at least over short distances (Bullock and Clarke, 2000; Jongejans and Telenius, 2001). Moreover, it relies on physical processes that can easily be translated into models (Tackenberg et al., 2003; Nathan et al., 2005; Soons and Ozinga, 2005). Although the first dispersal process, post-dispersal seed growth, and seed survival are what determine the distribution of species, these factors have been largely neglected (der Weduwen and Ruxton, 2019). Importantly, the distribution patterns of existing species reflect the geographic pattern of post-dispersal seed growth and survival. Some studies have focused on the distribution of a few samara species on the local scale (Tremblay et al., 2002) and for a few species (Wan et al., 2017), but few have explored the distribution of samara species using a large quantity of taxa data on a large scale. Therefore, to fully understand the distribution mechanism of samara species, it is necessary to analyze their broader geographic patterns and explore their climate drivers.

Understanding the factors driving the distribution of species is an important research area in ecology because it affects species coexistence and species diversity (Chesson, 2000). Abiotic constraints, dispersal limitations, and biotic interactions are the three main drivers that determine the distribution patterns of a species (Soberón, 2007). Moreover, phylogenetic conservatism explains the distribution of fruit types (Wang et al., 2021). These drivers of species distribution patterns act at different spatial scales (Kneitel and Chase, 2004), but climate is overall the most relevant variable when predicting continental to regional-scale plant species presence-absence distributions (Thuiller, 2004). This could explain why samara species are able to adapt to harsh environments. For example, the invasive samara species *Casuarina* is found in extreme abiotic conditions (sandy, nutrient-poor soils) and is tolerant to the extremes of soil moisture (from very dry to inundated). It can also tolerate sites with relatively high salinity, low soil fertility, and arid conditions. Besides this, most samara species frequently evolved in temperate regions (Mirle and Burnham, 1999) and, as a result, many species with samara fruits can withstand temperature variation in hot and cold climates. Furthermore, an essential, dry layer formed in samara fruits is easier to synthesize in drier environments (der Weduwen and Ruxton, 2019). Some samara species like *Ulmus minor* also have higher relatively drought tolerance (Guzmán-Delgado et al., 2017). However, a clear relationship between samara distribution on a broad scale and its environmental drivers has yet to be found.

Other traits like growth form could influence the distribution patterns of samara species and their relationship with environmental drivers. For instance, woody and herbaceous species are known to have different geographic distribution patterns (Hawkins et al., 2003). Woody species also need more moisture, while herbaceous species need more energy (Hawkins et al., 2003; Petit and Hampe, 2006). Moreover, growth form influences the dispersal distance (Thomson et al., 2018) and reproductive strategies of plants (Salguero-Gómez et al., 2016). Plant height increases across dispersal modes (unassisted < ant < vertebrate < wind; Thomson et al., 2018). Thus, woody samara species have a natural height advantage during primary dispersal compared to herbaceous species because release height is an important parameter in wind dispersal models (Tackenberg et al., 2003). Despite this, the influence of growth form on the relationship between samara species and climatic factors has remained poorly understood.

In this study, we examine the distribution patterns of woody and herbaceous samara species and assess how the proportion of samara species varies along climatic gradients across China. Specifically, we aim to: (1) examine the broad-scale geographical patterns of samara species in China; (2) evaluate the contribution of specific climatic variables to geographical patterns; and (3) assess whether geographic patterns and their determinants are consistent between different growth forms (woody and herbaceous species).

## Materials and Methods

### Species Distribution Data

Throughout the last 10 years, species distribution data was extracted from the Chinese Vascular Plant Distribution Database (Xu et al., 2018), which was assembled by the laboratory of Biodiversity and Biosafety in the Institute of Botany, the Chinese Academy of Sciences. The database was compiled from over 1,000 volumes of published books, inventory reports (Huang et al., 2016), and more than six million specimens (http://www.cvh.ac.cn/; Xu et al., 2018). Plant names were standardized using the Catalogue of Life China (Checklist 2015, http://www.sp2000.org.cn/) and the Flora of China.^1^ Cultivated and alien species were excluded from the dataset. Growth form information was identified based on the description of each species from the Flora of China. For each dry-fruited species, we classified the fruit type as winged or not winged. In total, there were 27,803 (11,222 woody and 16,581 herbaceous) species and 833 Samara (477 woody and 356 herbaceous) species that had both growth form and distribution data. The samara specie’s names and the growth form information was supplied in the supporting information in Supplementary Table S1. To avoid any influence from county size estimates and to reduce the effect of potential sampling incompleteness, we projected the species–county occurrence records to Albers equal-area grid cells with a resolution coarser than most counties (Xu et al., 2018). For this study, we transformed the county-level data to grid cells of 100 km × 100 km (Xu et al., 2018). We excluded grid cells with area <3,000 km^2^, leaving a total of 984 grid cells for our analyses. For each grid cell, we calculated the richness of the total occurred species, woody species and herbaceous species, respectively. Then, we calculated the richness of the total occurred samara species, woody samara species and herbaceous samara species. Finally, the proportion of samara species to total species in each grid cell was calculated.

### Environmental Factors

We used the temperature, precipitation, and climate variability variables as the main explanatory variables, since they are widely accepted roles in explaining biodiversity patterns. Wind speed was also included in this study due to its important role in samara species dispersal. Therefore, there are four kinds of climatic variables used in this study: (i) Precipitation variables, including annual precipitation (AP), precipitation of wettest quarter (PWQ) and mean precipitation of the driest quarter (PDQ); (ii) Temperature variables, including mean annual temperature (MAT), mean temperature of the coldest quarter (MTCQ) and temperature of the warmest quarter (TWQ); (iii) Climatic variability, including temperature seasonality (TS; standard deviation), precipitation seasonality (PS; coefficient of variation) and mean diurnal range [MDR, calculated as monthly mean × (maximum temperature − minimum temperature)]; (iv) Topographic data, including the altitude difference (TOP) and the Relief degree of the land surface (ARI); and (v) Wind speed data, including annual mean wind speed (AMeanW), annual maximum wind speed (AMaxW), and annual minimum wind speed (AMinW). All precipitation variables were square-root-transformed to improve the linearity and normality of the residuals. All the climate data used in this study came from the WorldClim 1.4 database with a resolution of 30 arc seconds.

In addition, we used the topographic data (TOP) and relief degree of the land surface (ARI) as explanatory variables for the samara species distribution. TOP was calculated as the elevation range within each grid cell using elevation data with a spatial resolution of 1 km^2^. The elevation data that was used to produce TOP was derived from the SRTM elevation data. ARI was extracted for each grid cell from China’s 1 km topographic relief data set (You et al., 2018).

### Statistical Analysis

To explore samara species geographic patterns, we performed the unitary ordinary least squares (OLS) linear regressions between the total, woody and herbaceous samara species richness/proportions in each grid cell and the geographic variables (latitude, longitude, and altitude). We also calculated the coefficient of determination (*R*^2^) to measure the importance of each geographic variable.

To explore the important drivers of the samara species geographic patterns, we first applied simple linear regression to study the relationship between single environmental factors and species proportions. We then applied a stepwise regression to explore the combined effects of environmental factors on samara proportions by the following steps. First, the best single predictor for the samara species proportion was kept in the model. Then all significant single factors were added to the model, and the Akaike Information Criterion (AIC) was applied to evaluate the quality of fit for the models. Finally, model Variance Inflation Factors (VIFs) were used to evaluate the model multicollinearity. Usually, model VIF should be smaller than 4 (Legendre and Legendre, 2012). If the model did not meet this criterion, we excluded one of the highly correlated variables in the multivariate analyses to avoid multicollinearity. Furthermore, the relative importance of the repressors in the final multiple OLS linear regression models was also calculated to measure the contribution of each variable to the final model. By taking a hierarchical approach, in which all orders of variables are used, the average independent contribution of a variable is obtained resulting in an exact partitioning (Chevan and Sutherland, 1991). To improve the robustness of the calculation, bootstrapping was performed 10,000 times to obtain the relative importance value intervals for each variable. Stepwise regression was conducted in R using the function “stepAIC” in the package MASS. VIFs were calculated using the function “VIF” in the package car. Relative importance values and bootstrapped intervals for each repressor were calculated using the function “boot.relimp” in the package relaimpo in R Core Team (2018).

However, residuals of all OLS models showed strong spatial autocorrelation, which could affect the significance test and bias parameter estimates. We used the simultaneous autoregressive error (SAR) model to account for spatial autocorrelation structure in the residuals of the model (Diniz-Filho et al., 2003). We tested SAR models with different neighborhood structures and spatial weights (lag distances were between 200 and 1,500 km). A SAR model with a lag distance of 300 km with a weighted neighborhood structure was the best spatial structure for total species and a lag distance of 400 km was best for the woody and herbaceous species. We used the Moran’s I statistic to determine if spatial autocorrelation was present in the residuals of the OLS and SAR models. The final model was selected based on the degree of reduction of spatial autocorrelation in the residuals and the minimization of AIC values. The expected Moran’s I value for low spatial autocorrelation is close to 0 (Dale and Fortin, 2014). Spatial statistics were performed with the package SPDEP in R (Bivand et al., 2013; Bivand and Piras, 2015).

## Results

### Spatial Patterns

**Figure 1:**
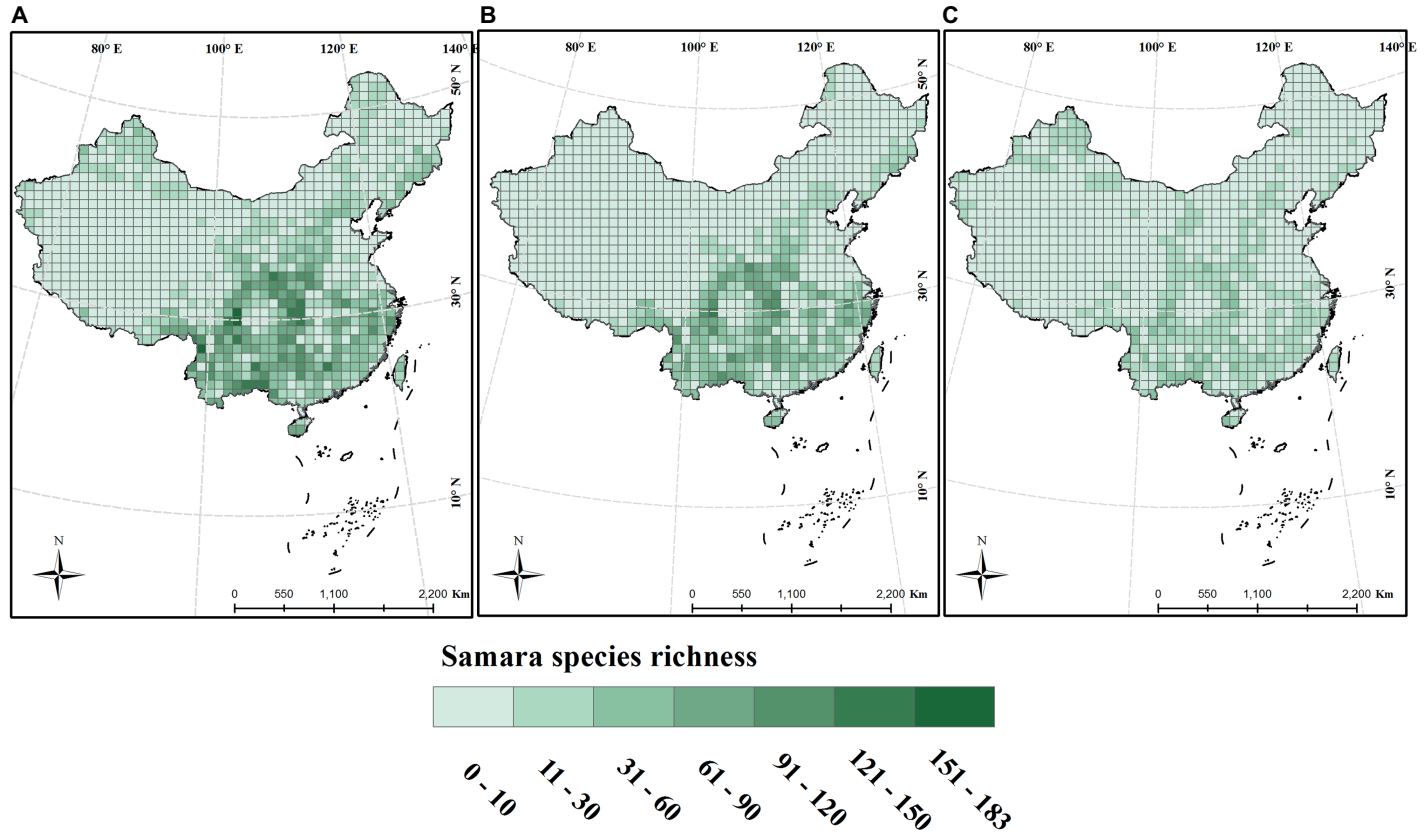
Distribution of samara species richness across China. Species richness was calculated within a 100 × 100 km grid. **(A)** Total species; **(B)** woody species; **(C)** herbaceous species.

**Figure 2:**
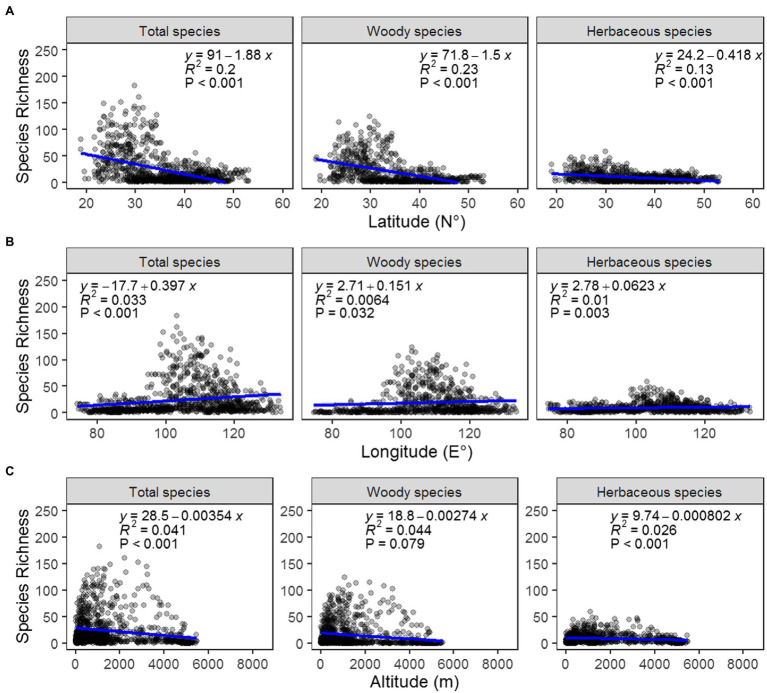
Regression analysis between the richness of total (left), woody (middle), and herbaceous (right) samara species and latitude **(A)**, longitude **(B)**, and altitude **(C)**.

From the analyses, samara species richness was highest in Southwest China within the lower latitude region. The maximum that was calculated for one grid was 124 species. The lowest was in the Tibetan Plateau, Xinjiang Region, with most of the grids containing less than 3 species (Figure 1A). Samara species richness was also higher in the central region around 100°E ~ 120°E and at an altitude between 500 and 1,500 m (Figures 1A, 2B,C). This geographic pattern is consistent for both the woody and the herbaceous species (Figures 1, 2). The species richness of woody samara species is relatively higher (the maximum density is 124 species per km) compared to the herbaceous species (the maximum density is 59 species per km; Figures 1B,C).

**Figure 3:**
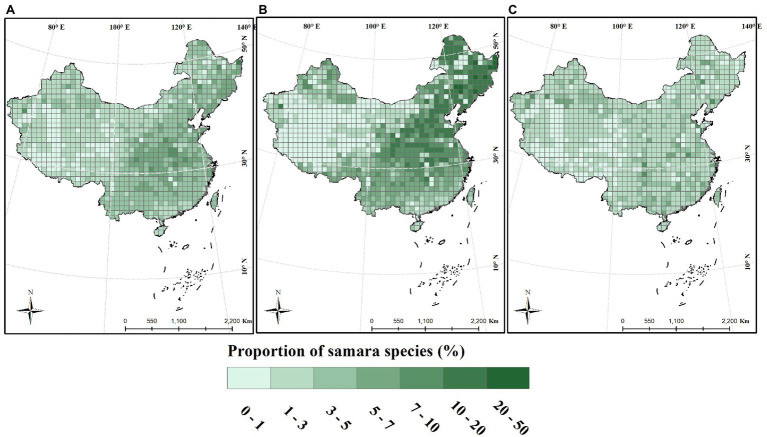
Distribution of the proportion of samara species across China. Species proportion was calculated within a 100 × 100 km grid. **(A)** Total species; **(B)** woody species; **(C)** herbaceous species.

**Figure 4:**
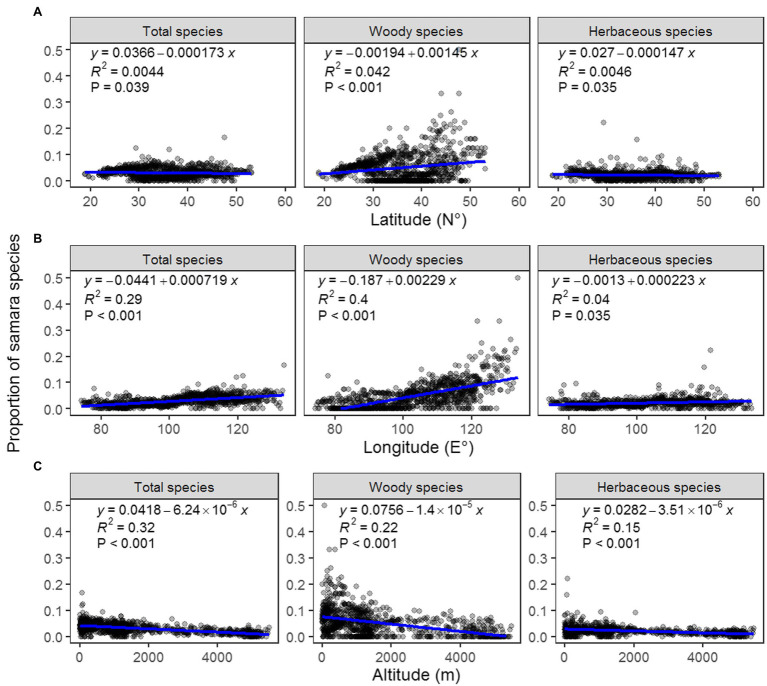
Regression analysis between the proportion of total (left), woody (middle), and herbaceous (right) samara species and latitude **(A)**, longitude **(B)**, and altitude **(C)**.

The proportion of woody samara species is highest in the North China Plain and Northeast China Plain. These locations are within the temperate monsoon region of Eastern China (Figure 3B). This geographic pattern was also supported by the regression analysis results: the proportion of woody samara species was positively correlated with longitude and negatively correlated with altitude (Figures 4B,C). Although the proportion of woody samara species was higher at higher latitude regions, this relationship was relatively poor compared to the longitude and altitude (Figure 3A). It was noted that the proportion of woody samara species is significantly higher than the herbaceous species (*p* < 0.05, Figures 3B,C). Specifically, the highest proportion for woody species is 50%, whereas the highest proportion for herbaceous species is 22% (Figures 3B,C). For the herbaceous samara species, the proportion did not show any clear latitude and longitude patterns (Figures 3C, 4A,B), but it did show similar altitudinal patterns as the woody species. The proportion of herbaceous samara species was higher at lower altitude regions (Figure 4C). For all the species considered, the proportion of samara species shared similar geographic patterns as the woody samara species (Figures 3, 4).

### Environmental Attributes

**Figure 5:**
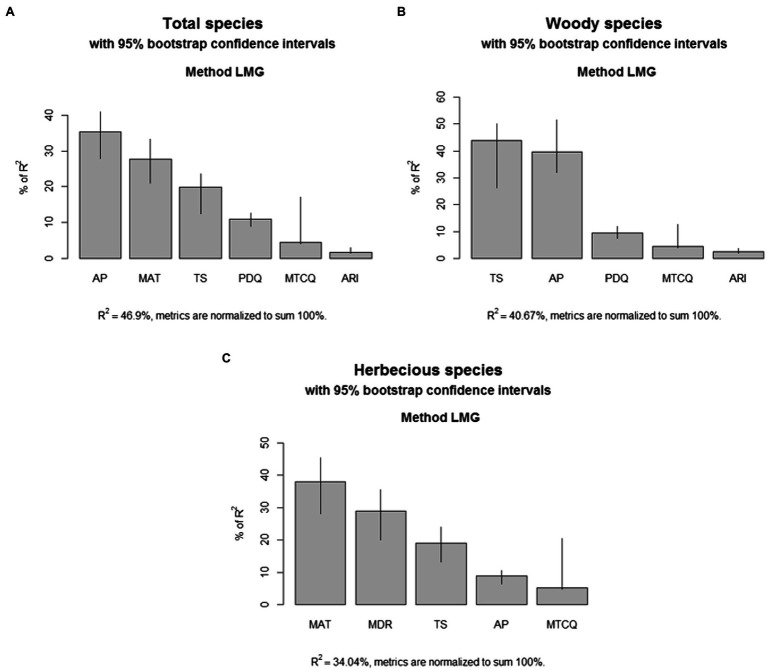
Relative importance of each climatic variable in the multi-linear models. **(A)** Total species; **(B)** woody species; **(C)** herbaceous species.

For total samara species, AP, TS, MAT, MTCQ, PDQ, and ARI were kept in the final model selection, and all these climatic factors together explain 46.91% of the variation in the geographic distribution of total samara species (*R^2^* = 0.469, Table 1). Based on the sign of the regression coefficients, the proportion of total samara species has a positive relationship with most of these environmental variables except for PDQ (Table 1). AP, MAT, and TS showed higher relative importance values compared with the other variables for the total samara species (Figure 5A). These three variables contribute 82.5% of the final model explanation for total samara species (Figure 5A).

**Table 1:** Results of multiple linear regression (OLS) models for all environmental variables and the proportion of total, woody, and herbaceous samara species to total species.

	*t*	VIF	Dev, %	AIC	Moran’s I
**All plants**					
Sqrt(AP)	14.66***	3.43	-	-	-
TS	16.44***	2.05	-	-	-
MAT	9.99***	2.26			
MTCQ	5.42***	1.73	-	-	-
Sqrt(PDQ)	−5.07***	3.17	-	-	-
ARI	3.22**	1.52	-	-	-
	-	-	46.91	1,271	0.219***
**Woody species**					
Sqrt(AP)	18.75***	3.37	-	-	-
TS	18.51***	2.54	-	-	-
Sqrt(PDQ)	−10.21***	3.05			
MTCQ	8.19***	1.68			
ARI	2.43**	1.41	-	-	-
	-	-	40.67	1,275	0.312***
**Herbaceous species**					
MAT	8.74***	2.36	-	-	-
TS	9.92***	1.63	-	-	-
MDR	−8.18***	3.66	-	-	-
Sqrt(AP)	−3.97***	3.65	-	-	-
MTCQ	3.11**	1.59	-	-	-
	-	-	34.03	1,095	0.098***

***p < 0.001, **0.001 < *p* < 0.01, *0.01 < *p* < 0.05.

*AP, mean annual precipitation; MAT, mean annual temperature; TS, temperature seasonality; MTCQ, the temperature of the coldest quarter; MDR, mean diurnal range; ARI, relief degree of land surface; PDQ, precipitation of the driest quarter; unique-R^2^, differences between the R^2^ of full MLR models and that of MLR models without that predictor; VIF, variance inflation factor, used to evaluate the significance of multi-collinearity; Dev, percentage deviance explained by the models; ‘-’, no value. If VIF is greater than five, then multi-collinearity is considered significant. Significance was determined using Student’s t-test (α = 0.05)*.

For woody samara species, AP, TS, PDQ, MTCQ, and ARI were kept in the final model selection and all these climatic factors together explain 40.67% of the variation in the geographic distribution of woody samara species (*R^2^* = 0.469, Table 1). Based on the sign of the regression coefficients, the proportion of woody samara species has a positive relationship with most of these environmental variables except for PDQ (Table 1). This means that woody samara species preferred to inhabit areas where the climate is wet and mild, but temperature seasonality and the relief degree of the land surface are high (Table 1; Figures 1, 3B). TS and AP had the most effect on the distribution of woody samara species (Figure 5B). These two variables contribute 83.6% of the final model explanation for the woody samara species (Figure 5B).

For the herbaceous samara species, MAT, TS, MDR, AP, and MTCQ were kept in the final model selection, and all these factors explain 34.03% of the variation in the distribution of herbaceous samara species (Table 1). Based on the sign of the regression coefficients, the proportion of herbaceous samara species positively correlated with MAT, TS, and MTCQ and negatively correlated with MDR and AP (Table 1). Among these climatic factors, MAT, MDR, and TS (all temperature related factors) contribute the most to the distribution of the herbaceous samara species (Figure 5C). These three variables contribute 86.7% of the final model explanation for the herbaceous samara species (Figure 5B).

The VIFs of the final models are all smaller than 4, indicating that the regressors of these multi-linear regression models have little multicollinearity and that the results of the models are reliable. Their Moran’s I statistics are significantly greater than zero, indicating the multi-linear regression models have spatial autocorrelation. This spatial autocorrelation was removed from the simultaneous autoregressive error (SAR) models. Afterwards, all these models shared similar results with the OLS models and improved models Pseudo-*R*^2^ (Table 2).

**Table 2:** Results of simultaneous autoregressive error (SAR) models for all environmental variables and the proportion of total, woody, and herbaceous samara species to total species.

	*Z*	Pseudo-*R*^2^	AIC	Moran’s I
**All plants**				
Sqrt(AP)	2.34*	-	-	-
TS	1.41*	-	-	-
MTCQ	1.04	-	-	-
MAT	2.89**	-	-	-
ARI	2.07*	-	-	-
Sqrt(PDQ)	−0.15	-	-	-
-	-	60.78	994	−0.007
**Woody species**				
Sqrt(AP)	3.92***	-	-	-
TS	3.12**	-	-	-
MTCQ	2.53*	-	-	-
PDQ	−0.78	-	-	-
ARI	1.81*	-	-	-
-	-	60.48	985	−0.009
**Herbaceous species**				
MAT	4.17***	-	-	-
TS	3.06**	-	-	-
MDR	−4.41***	-	-	-
MTCQ	2.16*	-	-	-
Sqrt(AP)	−1.03	-	-	-
-	-	39.84	1,015	−0.003

***p < 0.001, **0.001 < *p* < 0.01, *0.01 < *p* < 0.05.

*The proportion of samara species was log-transformed for the analysis. All the p-values of the Moran’s I tests for the SAR models were greater than 0.1. AP, mean annual precipitation (was square-root transformed in the analysis). MAT, mean annual temperature; TS, temperature seasonality; MTCQ, the temperature of the coldest quarter; MDR, mean diurnal range; ARI, relief degree of land surface; PDQ, precipitation of the driest quarter; pseudo-R^2^, the squared Pearson correlation coefficient comparing the observed and predicted proportions of samara species using full models; ‘-’, no value. Significance was determined using Student’s t-test (α = 0.05)*.

## Discussion

### Geographical Patterns of Samara Species Distribution

The patterns of biodiversity and the mechanisms that cause these patterns, are a primary focus of biodiversity research (Gaston, 2000). We explored the distribution patterns of samara species with different growth forms and found that the richness of samara species is highest in the mountainous areas of southern China like the Hengduan Mountains (Figure 2). This is consistent with the distribution of endangered plant species diversity in China (Tang et al., 2006; Zhang et al., 2015; Huang et al., 2016). History, topography, and environmental factors shape this geographic pattern. Geographically, this area contains subtropical evergreen broad-leaved forests, tropical monsoon forests, and vegetational rain forests. These all have high levels of physiognomic heterogeneity, older, more established plant populations, and more complexity than other areas in China (Geography, 1985). The mountainous areas of southern China offer a diversity of environments that contribute greatly to the survival, speciation, and evolution of vascular plants in China (Axelrod et al., 1996; Qian, 2002). The mountainous regions provide rich habitats for much of the country’s remaining biodiversity, owing to the heterogeneity of climates and soils, rapid elevational changes, varying aspects of slope direction, abundant microhabitats, and limited suitability for cultivation (Korner and Spehn, 2019). Fortunately, China has established more than 2,000 national and local nature reserves, 73.8% of which are in mountainous regions. These reserves cover a total area of 1.5 million km^2^ (83.0% of all protected areas in China; Tang et al., 2006). Therefore, samara species diversity will benefit from policies that protect mountainous regions.

The proportion distribution of samara species reflected their preference for certain habitats and their close relationship with environmental factors. In contrast with fleshy-fruited species that tend to grow in the tropical and subtropical regions of southern China (Zhao et al., 2018), samara species prefer the temperate regions of central and northeastern China (Figure 3). This region has a temperate monsoon climate characterized by the dry, cold winters and wet, warm summers. This result is consistent with the previous finding that wind-dispersed trees are common in temperate and boreal forests (Bonner, 2008). Samaras fruits have a low moisture content, which gives them ability to prevent freezing damage at low temperatures in temperate regions. For instance, high tolerance to low-temperature stress was found in Siberian maple (Pan et al., 2009). Furthermore, the drier winter in temperate regions is beneficial to samara maturation, since most wind-dispersed species produce fruits during the dry season when wind intensity is highest and humidity is lowest (Jara-Guerrero et al., 2011). In the temperate regions of China, the alien invasive species richness is higher in eastern China (Zhou et al., 2020). Samara, as an invasive species, would have an advantage in distribution range expansion since they can easily disperse and have a high tolerance to harsh environments, especially in temperate regions. Thus, it is worthy to explore the fruit type and dispersal modes of invasive species. This information could be helpful for evaluating their potential threats and distribution patterns.

The woody growth form has a higher proportion of samara species than the herbaceous growth form (Figures 3B,C). This is consistent with studies conducted on local forest communities (Jara-Guerrero et al., 2011). Plant growth forms are closely related to the life-history, survival strategies, and dispersal traits (Beckman et al., 2018). Trees tend to be the tallest plant species as plant height increases across growth forms (Moles et al., 2009). Plant height, for example, is a very important trait that influences dispersal distance (Thomson et al., 2018), especially for the wind-dispersal species. Woody species could have a higher proportion of samara species than the herbaceous species because taller plants are more likely to invest in the presence of dispersal structures and have higher dispersal ability compared to herbaceous species (Beckman et al., 2018). Indeed, when fruiting type is considered, woody species tend to invest more energy in their dispersal structure, e.g., more samara fruits (in this study) or fleshy fruits (Zhao et al., 2018), which could improve their seed dispersal ability relative to herbaceous species.

### Environmental Attributes

For the woody samara species, annual precipitation, and temperature seasonality are the top two factors contributing to the shape of the species’ geographic patterns, whereas mean temperature, mean temperature diurnal range, and temperature seasonality are the most important drivers of herbaceous samara species’ geographic patterns (Tables 1, 2). In addition, the proportion of herbaceous samara species show a negative response to annual precipitation compared with the positive response that the woody samara species have (Tables 1, 2). These results indicate that growth form can influence the relationship between samara fruit traits and climate. Differences in functional adaptations to environmental conditions between these two groups have also been identified before (Petit and Hampe, 2006; Ordoñez et al., 2010; Šímová et al., 2018). For example, woody species were reported to have larger fruits and heights and, as a result, may need more water to transport nutrients. Herbaceous species, on the other hand, may acquire energy more rapidly to accumulate biomass for organ growth due to their short lifetime (Zhao et al., 2018). The growth of woody plants is more sensitive to spatial variation in rainfall than the herbaceous plants (Kadmon and Danin, 1999). Herbaceous plants, on the other hand, generally dominate at higher latitudes, and their growth is more highly correlated with energy variables than that of woody plants (Hawkins et al., 2003). In addition, woody samara species distribute in areas with a higher land surface roughness when compared with herbaceous species (Tables 1, 2). This may be due to the fruits of woody species having a height advantage over herbaceous samara fruits on rough land surfaces (Wright et al., 2008). The increase in the roughness of the land surface could also increase the amount of updraft, which is considered important for long-distance seed dispersal (Nathan and Katul, 2005; Kuparinen, 2006).

Woody and herbaceous samara species both have a positive relationship with temperature seasonality (Tables 1, 2). This indicates that samara species are better adapted to living in places with a higher temperature seasonality. Samara fruits have a low moisture content and, therefore, may be able to withstand lower temperatures and/or greater variations in temperature. For example, *Pinus* spp. seeds have larger wings in environments with greater temperature variability and/or are prone to fire (Salazar-Tortosa et al., 2019) and wind dispersal occurs more frequently in taxa adapted to climates where temperature (but not precipitation) is highly variable (Salazar-Tortosa et al., 2019). In addition, woody and herbaceous samara species have a negative relationship with dry season precipitation and annual precipitation, respectively (Tables 1, 2). This indicates that the samara species prefer to grow in drier places. This might be related to the characteristics of samara fruits. During development, samaras develop abscission layers that allow the release of the samara under the right meteorological conditions (Bohrer et al., 2008). The abscission layers form faster under less humid conditions and if humidity is low enough the layers can form within 3 h (Greene and Johnson, 1992).

Empirical studies have shown that wind is a common dispersal agent of seeds and pollen, and that wind speed is an important driver of wind dispersal (Bullock and Clarke, 2000; Bullock et al., 2012). Surprisingly, wind speed is not an important driver in determining the distribution of samara species in China at the regional scale (Figure 4). This contradicts previous studies conducted at individual and community scales. For example, wind (an external factors) interacting with plant traits (an internal factor) determined the distribution of wind-dispersed species within communities in one study (Wright et al., 2008). Wind speed and direction were also key parameters in wind dispersal models (Wan et al., 2017). Three factors may have caused this result. First, wind dispersal usually happens during the process of primary dispersal, whereas the secondary dispersal process can greatly improve their distance. This is very common in urban settings for the invasive samara species since there are many urban road corridors (Kowarik and von der Lippe, 2011). In the North China Plain and Northeast China Plain, the highly disturbed urban settings provide samara species with greater secondary dispersal distances and thus improve the possibility of diffusion into suitable environments. Secondly, the massive number of species that this study analyzed could have obscured the response of individual samara species to wind. Thirdly, the diverse biome types in China could have masked the influence of wind on the distribution of samara species. For example, tropical and subtropical biomes have a negative response to wind, while boreal and temperate biomes have a positive response (Wan et al., 2017).

Species distribution and ecological niche models have been widely used to study the distribution of many taxa (Huettmann et al., 2011; Padalia et al., 2014; Wan et al., 2017). However, most papers that use these models focus on less than 10 species at a time (Dyderski et al., 2018). Since different species usually have divergent responses to environmental change, it is difficult to build a cross-species distribution model. Given the large number of samara species and the massive county level presence records used in this study, we only used the regression analysis to examine the relationship between the distribution of samara species and a few important environmental variables. This method was simple and provided us with a clear relationship between the richness/proportion of samara species and each environmental variable. We believe that using samara species distribution models to evaluate the risk of invasive samara species is a promising direction for our research. In addition, we should note that our findings from this study could be biased by the uncompleted species distribution records in the real world, since some species’ areas were not accessible due to geographic boundaries. To solve this problem, samara species should be recorded at the continental scale or even globally. International species distribution databases, such as, Global Biodiversity Information Facility (GBIF, 2017) and the EUFORGEN database,^2^ make it possible to solve this problem in the future.

For the first time, our study provides a clear geographic distribution pattern of samara. The data shows that woody samara species tend to be distributed in the temperate monsoon regions (Figure 3). Moreover, precipitation, temperature, and temperature seasonality contribute heavily to the distribution of samara species (Figure 4). This information could be used to predict distribution models of samara species under future climate conditions. In this study, we determined that the temperature of the central and northeastern temperate regions of China are continuing to rise (Ding et al., 2007) and drought is becoming severe (Piao et al., 2010). Importantly, our study shows that samara species with different growth forms have different responses to climatic factors. This indicates that plant growth form is an important input variable for modeling the habitat distribution of samara species. In the future, the impact of other abiotic variables, including the human footprint, soil factors, and biome types should also be explored using environmental niche modeling to project the habitat distribution of samara species (Hoffman et al., 2008; Padalia et al., 2014; Gallardo et al., 2015).

## Conclusion

In summary, we assessed the biogeographic patterns of samara species and the underlying environmental drivers among the different growth forms of samaras in the tropical and temperate zones of China. Our results show that samara species richness is higher in southwest regions and that the proportion of woody samara species is high in the temperate monsoon China. The proportion of woody samara species is higher than the herbaceous samara species overall. Results also suggest that these patterns are driven by large-scale temperature and precipitation gradients. Temperature seasonality and annual precipitation are the two most important factors involved in the distribution of woody samara species, whereas mean temperature, mean temperature diurnal range, temperature seasonality are the most important drivers of herbaceous samara species distribution. This work advances our understanding of the relationships between the different growth forms of samara species and several environmental gradients. It also provides a basis and a reference for the influence of climate change on the distribution of samara species.

## Data Availability Statement

The datasets presented in this study can be found in online repositories. The names of the repository/repositories and accession number(s) can be found in the article/Supplementary Material.

## Author Contributions

YD conceived the study. YZ performed the analysis and wrote the paper. SD, GC, and KM provide the datasets. XW and YD provided feedback as the paper took form and commented on draft versions of the paper. All authors contributed to the article and approved the submitted version.

## Funding

This work was supported by the National Specimen Information Infrastructure of China and supported by the Hainan University (KYQD(ZR) 1979).

## Conflict of Interest

The authors declare that the research was conducted in the absence of any commercial or financial relationships that could be construed as a potential conflict of interest.

## Publisher’s Note

All claims expressed in this article are solely those of the authors and do not necessarily represent those of their affiliated organizations, or those of the publisher, the editors and the reviewers. Any product that may be evaluated in this article, or claim that may be made by its manufacturer, is not guaranteed or endorsed by the publisher.

## Footnotes

^1^ http://foc.eflora.cn/

^2^ http://www.euforgen.org/distribution_maps.html
