# Supplementary material for: The Diversity Distribution and Climatic Niche of Samara Species in China
Source: Front Plant Sci. 2022 Jun 17;13:895720. doi: 10.3389/fpls.2022.895720 (PMC9249021; doi:10.3389/fpls.2022.895720)
Supplement: Supplementary file 1 [file Table_1.DOCX]

| **Species names** | **Growth form** |
| --- | --- |
| Acer acuminatum | woody |
| Acer acutum | woody |
| Acer albopurpurascens | woody |
| Acer amplum | woody |
| Acer amplum subsp. bodinieri | woody |
| Acer amplum subsp. catalpifolium | woody |
| Acer amplum subsp. tientaiense | woody |
| Acer barbinerve | woody |
| Acer buergerianum | woody |
| Acer buergerianum var. formosanum | woody |
| Acer buergerianum var. horizontale | woody |
| Acer buergerianum var. jiujiangense | woody |
| Acer buergerianum var. kaiscianense | woody |
| Acer buergerianum var. yentangense | woody |
| Acer caesium | woody |
| Acer campbellii | woody |
| Acer campbellii var. serratifolium | woody |
| Acer cappadocicum | woody |
| Acer cappadocicum var. sinicum | woody |
| Acer caudatifolium | woody |
| Acer caudatum | woody |
| Acer chienii | woody |
| Acer chingii | woody |
| Acer chunii | woody |
| Acer chunii subsp. dimorphophyllum | woody |
| Acer confertifolium | woody |
| Acer cordatum | woody |
| Acer cordatum var. dimorphifolium | woody |
| Acer crassum | woody |
| Acer davidii | woody |
| Acer davidii subsp. grosseri | woody |
| Acer duplicatoserratum | woody |
| Acer duplicatoserratum var. chinense | woody |
| Acer elegantulum | woody |
| Acer erianthum | woody |
| Acer fabri | woody |
| Acer fenzelianum | woody |
| Acer flabellatum | woody |
| Acer forrestii | woody |
| Acer fulvescens | woody |
| Acer gracilifolium | woody |
| Acer griseum | woody |
| Acer henryi | woody |
| Acer hilaense | woody |
| Acer kungshanense | woody |
| Acer kwangnanense | woody |
| Acer kweilinense | woody |
| Acer laevigatum | woody |
| Acer laevigatum var. salweenense | woody |
| Acer laurinum | woody |
| Acer laxiflorum | woody |
| Acer leipoense | woody |
| Acer linganense | woody |
| Acer longipes | woody |
| Acer lucidum | woody |
| Acer lungshengense | woody |
| Acer mandshuricum | woody |
| Acer maximowiczianum | woody |
| Acer maximowiczii | woody |
| Acer metcalfii | woody |
| Acer miaoshanicum | woody |
| Acer miaotaiense | woody |
| Acer morrisonense | woody |
| Acer oblongum | woody |
| Acer oblongum var. omeiense | woody |
| Acer oligocarpum | woody |
| Acer oliverianum | woody |
| Acer paihengii | woody |
| Acer palmatum | woody |
| Acer pauciflorum | woody |
| Acer paxii | woody |
| Acer pectinatum | woody |
| Acer pectinatum subsp. taronense | woody |
| Acer pentaphyllum | woody |
| Acer pictum | woody |
| Acer pictum subsp. macropterum | woody |
| Acer pictum subsp. mono | woody |
| Acer pictum subsp. pubigerum | woody |
| Acer pictum subsp. tricuspis | woody |
| Acer pilosum | woody |
| Acer pilosum var. stenolobum | woody |
| Acer pinnatinervium | woody |
| Acer poliophyllum | woody |
| Acer pseudosieboldianum | woody |
| Acer pubinerve | woody |
| Acer pubipetiolatum | woody |
| Acer pubipetiolatum var. pingpienense | woody |
| Acer saccharinum | woody |
| Acer semenovii | woody |
| Acer serrulatum | woody |
| Acer shenkanense | woody |
| Acer shihweii | woody |
| Acer sikkimense | woody |
| Acer sinense | woody |
| Acer sino oblongum | woody |
| Acer sinopurpurascens | woody |
| Acer stachyophyllum | woody |
| Acer stachyophyllum subsp. tetramerum | woody |
| Acer sterculiaceum | woody |
| Acer sterculiaceum subsp. franchetii | woody |
| Acer sutchuenense | woody |
| Acer sycopseoides | woody |
| Acer tataricum | woody |
| Acer tataricum subsp. ginnala | woody |
| Acer tegmentosum | woody |
| Acer tenellum | woody |
| Acer tenellum var. septemlobum | woody |
| Acer thomsonii | woody |
| Acer tibetense | woody |
| Acer tonkinense | woody |
| Acer triflorum | woody |
| Acer truncatum | woody |
| Acer tschonoskii | woody |
| Acer tschonoskii subsp. koreanum | woody |
| Acer tsinglingense | woody |
| Acer tutcheri | woody |
| Acer ukurundense | woody |
| Acer wangchii | woody |
| Acer wardii | woody |
| Acer wilsonii | woody |
| Acer yinkunii | woody |
| Acer yui | woody |
| Ailanthus altissima | woody |
| Ailanthus altissima var. sutchuenensis | woody |
| Ailanthus altissima var. tanakai | woody |
| Ailanthus fordii | woody |
| Ailanthus giraldii | woody |
| Ailanthus triphysa | woody |
| Ailanthus vilmoriniana | woody |
| Alnus cremastogyne | woody |
| Alnus ferdinandi coburgii | woody |
| Alnus formosana | woody |
| Alnus hirsuta | woody |
| Alnus japonica | woody |
| Alnus lanata | woody |
| Alnus mandshurica | woody |
| Alnus nepalensis | woody |
| Alnus trabeculosa | woody |
| Ancistrocladus tectorius | woody |
| Angelica amurensis | herbaceous |
| Angelica anomala | herbaceous |
| Angelica apaensis | herbaceous |
| Angelica balangshanensis | herbaceous |
| Angelica biserrata | herbaceous |
| Angelica cartilaginomarginata | herbaceous |
| Angelica cartilaginomarginata var. foliosa | herbaceous |
| Angelica cincta | herbaceous |
| Angelica dahurica | herbaceous |
| Angelica dahurica var. formosana | herbaceous |
| Angelica decursiva | herbaceous |
| Angelica dielsii | herbaceous |
| Angelica duclouxii | herbaceous |
| Angelica fargesii | herbaceous |
| Angelica gigas | herbaceous |
| Angelica kangdingensis | herbaceous |
| Angelica laxifoliata | herbaceous |
| Angelica likiangensis | herbaceous |
| Angelica longicaudata | herbaceous |
| Angelica longipedicellata | herbaceous |
| Angelica longipes | herbaceous |
| Angelica maowenensis | herbaceous |
| Angelica megaphylla | herbaceous |
| Angelica morii | herbaceous |
| Angelica morrisonicola | herbaceous |
| Angelica morrisonicola var. nanhutashanensis | herbaceous |
| Angelica multicaulis | herbaceous |
| Angelica nitida | herbaceous |
| Angelica omeiensis | herbaceous |
| Angelica oncosepala | herbaceous |
| Angelica paeoniifolia | herbaceous |
| Angelica pinnatiloba | herbaceous |
| Angelica polymorpha | herbaceous |
| Angelica pseudoselinum | herbaceous |
| Angelica setchuenensis | herbaceous |
| Angelica sinensis | herbaceous |
| Angelica songpanensis | herbaceous |
| Angelica sylvestris | herbaceous |
| Angelica ternata | herbaceous |
| Angelica tsinlingensis | herbaceous |
| Angelica valida | herbaceous |
| Anogeissus acuminata | woody |
| Archangelica brevicaulis | herbaceous |
| Archangelica decurrens | herbaceous |
| Arcuatopterus linearifolius | herbaceous |
| Arcuatopterus sikkimensis | herbaceous |
| Arcuatopterus thalictrioideus | herbaceous |
| Aspidopterys cavaleriei | woody |
| Aspidopterys concava | woody |
| Aspidopterys esquirolii | woody |
| Aspidopterys floribunda | woody |
| Aspidopterys glabriuscula | woody |
| Aspidopterys henryi | woody |
| Aspidopterys microcarpa | woody |
| Aspidopterys nutans | woody |
| Aspidopterys obcordata | woody |
| Aspidopterys obcordata var. hainanensis | woody |
| Atriplex aucheri | herbaceous |
| Atriplex cana | herbaceous |
| Atriplex centralasiatica | herbaceous |
| Atriplex dimorphostegia | herbaceous |
| Atriplex fera | herbaceous |
| Atriplex laevis | herbaceous |
| Atriplex maximowicziana | herbaceous |
| Atriplex prostrata | herbaceous |
| Atriplex repens | woody |
| Atriplex sibirica | herbaceous |
| Atriplex tatarica | herbaceous |
| Atriplex verrucifera | woody |
| Begonia acutitepala | herbaceous |
| Begonia algaia | herbaceous |
| Begonia alveolata | herbaceous |
| Begonia arboreta | herbaceous |
| Begonia asperifolia | herbaceous |
| Begonia asperifolia var. tomentosa | herbaceous |
| Begonia asperifolia var. unialata | herbaceous |
| Begonia augustinei | herbaceous |
| Begonia baviensis | herbaceous |
| Begonia biflora | herbaceous |
| Begonia cathayana | herbaceous |
| Begonia cavaleriei | herbaceous |
| Begonia ceratocarpa | herbaceous |
| Begonia chingii | herbaceous |
| Begonia chishuiensis | herbaceous |
| Begonia chitoensis | herbaceous |
| Begonia circumlobata | herbaceous |
| Begonia cirrosa | herbaceous |
| Begonia clavicaulis | herbaceous |
| Begonia crystallina | herbaceous |
| Begonia cucurbitifolia | herbaceous |
| Begonia daweishanensis | herbaceous |
| Begonia daxinensis | herbaceous |
| Begonia dielsiana | herbaceous |
| Begonia digyna | herbaceous |
| Begonia discreta | herbaceous |
| Begonia dryadis | herbaceous |
| Begonia duclouxii | herbaceous |
| Begonia emeiensis | herbaceous |
| Begonia fangii | herbaceous |
| Begonia fenicis | herbaceous |
| Begonia filiformis | herbaceous |
| Begonia fimbristipula | herbaceous |
| Begonia flaviflora | herbaceous |
| Begonia flaviflora var. vivida | herbaceous |
| Begonia fordii | herbaceous |
| Begonia formosana | herbaceous |
| Begonia forrestii | herbaceous |
| Begonia gagnepainiana | herbaceous |
| Begonia glechomifolia | herbaceous |
| Begonia grandis | herbaceous |
| Begonia grandis subsp. holostyla | herbaceous |
| Begonia grandis var. sinensis | herbaceous |
| Begonia grandis var. unialata | herbaceous |
| Begonia guangxiensis | herbaceous |
| Begonia guishanensis | herbaceous |
| Begonia gulinqingensis | herbaceous |
| Begonia gungshanensis | herbaceous |
| Begonia hainanensis | herbaceous |
| Begonia handelii | herbaceous |
| Begonia handelii var. prostrata | herbaceous |
| Begonia handelii var. rubropilosa | herbaceous |
| Begonia hatacoa | herbaceous |
| Begonia hekouensis | herbaceous |
| Begonia hemsleyana | herbaceous |
| Begonia hemsleyana var. kwangsiensis | herbaceous |
| Begonia henryi | herbaceous |
| Begonia howii | herbaceous |
| Begonia huangii | herbaceous |
| Begonia hymenocarpa | herbaceous |
| Begonia imitans | herbaceous |
| Begonia jingxiensis | herbaceous |
| Begonia josephii | herbaceous |
| Begonia labordei | herbaceous |
| Begonia lacerata | herbaceous |
| Begonia laminariae | herbaceous |
| Begonia lanternaria | herbaceous |
| Begonia limprichtii | herbaceous |
| Begonia lipingensis | herbaceous |
| Begonia lithophila | herbaceous |
| Begonia longicarpa | herbaceous |
| Begonia longifolia | herbaceous |
| Begonia longistyla | herbaceous |
| Begonia lukuana | herbaceous |
| Begonia macrotoma | herbaceous |
| Begonia malipoensis | herbaceous |
| Begonia manhaoensis | herbaceous |
| Begonia masoniana | herbaceous |
| Begonia megalophyllaria | herbaceous |
| Begonia mengtzeana | herbaceous |
| Begonia miranda | herbaceous |
| Begonia modestiflora | herbaceous |
| Begonia morifolia | herbaceous |
| Begonia morsei | herbaceous |
| Begonia muliensis | herbaceous |
| Begonia ningmingensis | herbaceous |
| Begonia obliquifolia | herbaceous |
| Begonia obsolescens | herbaceous |
| Begonia oreodoxa | herbaceous |
| Begonia ornithophylla | herbaceous |
| Begonia palmata | herbaceous |
| Begonia palmata var. bowringiana | herbaceous |
| Begonia palmata var. crassisetulosa | herbaceous |
| Begonia palmata var. difformis | herbaceous |
| Begonia palmata var. laevifolia | herbaceous |
| Begonia parvula | herbaceous |
| Begonia paucilobata | herbaceous |
| Begonia paucilobata var. maguanensis | herbaceous |
| Begonia pedatifida | herbaceous |
| Begonia peii | herbaceous |
| Begonia peltatifolia | herbaceous |
| Begonia picta | herbaceous |
| Begonia platycarpa | herbaceous |
| Begonia polytricha | herbaceous |
| Begonia porteri | herbaceous |
| Begonia pseudodryadis | herbaceous |
| Begonia psilophylla | herbaceous |
| Begonia purpureofolia | herbaceous |
| Begonia ravenii | herbaceous |
| Begonia rex | herbaceous |
| Begonia rhynchocarpa | herbaceous |
| Begonia rockii | herbaceous |
| Begonia rotundilimba | herbaceous |
| Begonia ruboides | herbaceous |
| Begonia scitifolia | herbaceous |
| Begonia setifolia | herbaceous |
| Begonia sikkimensis | herbaceous |
| Begonia sinofloribunda | herbaceous |
| Begonia smithiana | herbaceous |
| Begonia subhowii | herbaceous |
| Begonia suboblata | herbaceous |
| Begonia summoglabra | herbaceous |
| Begonia taiwaniana | herbaceous |
| Begonia taliensis | herbaceous |
| Begonia truncatiloba | herbaceous |
| Begonia umbraculifolia | herbaceous |
| Begonia versicolor | herbaceous |
| Begonia villifolia | herbaceous |
| Begonia wangii | herbaceous |
| Begonia wenshanensis | herbaceous |
| Begonia yingjiangensis | herbaceous |
| Begonia yui | herbaceous |
| Begonia zhengyiana | herbaceous |
| Berrya cordifolia | woody |
| Betula albosinensis | woody |
| Betula alnoides | woody |
| Betula austrosinensis | woody |
| Betula calcicola | woody |
| Betula chinensis | woody |
| Betula costata | woody |
| Betula cylindrostachya | woody |
| Betula dahurica | woody |
| Betula delavayi | woody |
| Betula delavayi var. microstachya | woody |
| Betula delavayi var. polyneura | woody |
| Betula ermanii | woody |
| Betula ermanii var. macrostrobila | woody |
| Betula ermanii var. yingkiliensis | woody |
| Betula fargesii | woody |
| Betula fruticosa | woody |
| Betula gmelinii | woody |
| Betula gynoterminalis | woody |
| Betula halophila | woody |
| Betula humilis | woody |
| Betula insignis | woody |
| Betula jinpingensis | woody |
| Betula jiulungensis | woody |
| Betula luminifera | woody |
| Betula microphylla | woody |
| Betula middendorfii | woody |
| Betula ovalifolia | woody |
| Betula pendula | woody |
| Betula platyphylla | woody |
| Betula platyphylla var. phellodendroides | woody |
| Betula potaninii | woody |
| Betula rhombibracteata | woody |
| Betula rotundifolia | woody |
| Betula schmidtii | woody |
| Betula tianschanica | woody |
| Betula trichogemma | woody |
| Betula utilis | woody |
| Boniodendron minius | woody |
| Burretiodendron esquirolii | woody |
| Burretiodendron kydiifolium | woody |
| Calligonum aphyllum | woody |
| Calligonum leucocladum | woody |
| Calligonum rubicundum | woody |
| Callitriche fehmedianii | herbaceous |
| Callitriche hermaphroditica | herbaceous |
| Callitriche japonica | herbaceous |
| Callitriche palustris | herbaceous |
| Callitriche palustris var. elegans | herbaceous |
| Callitriche stagnalis | herbaceous |
| Camptotheca acuminata | woody |
| Cardiopteris quinqueloba | herbaceous |
| Carpinus chuniana | woody |
| Carpinus cordata | woody |
| Carpinus cordata var. chinensis | woody |
| Carpinus cordata var. mollis | woody |
| Carpinus dayongina | woody |
| Carpinus fangiana | woody |
| Carpinus fargesiana | woody |
| Carpinus fargesiana var. hwai | woody |
| Carpinus firmifolia | woody |
| Carpinus glanduloso punctata | woody |
| Carpinus hebestroma | woody |
| Carpinus henryana | woody |
| Carpinus hupeana | woody |
| Carpinus kawakamii | woody |
| Carpinus kweichowensis | woody |
| Carpinus londoniana | woody |
| Carpinus londoniana var. lanceolata | woody |
| Carpinus londoniana var. xiphobracteata | woody |
| Carpinus microphylla | woody |
| Carpinus minutiserrata | woody |
| Carpinus mollicoma | woody |
| Carpinus monbeigiana | woody |
| Carpinus oblongifolia | woody |
| Carpinus omeiensis | woody |
| Carpinus polyneura | woody |
| Carpinus pubescens | woody |
| Carpinus purpurinervis | woody |
| Carpinus putoensis | woody |
| Carpinus rankanensis | woody |
| Carpinus rankanensis var. matsudae | woody |
| Carpinus rupestris | woody |
| Carpinus shensiensis | woody |
| Carpinus stipulata | woody |
| Carpinus sungpanensis | woody |
| Carpinus tientaiensis | woody |
| Carpinus tsaiana | woody |
| Carpinus tschonoskii | woody |
| Carpinus tsunyihensis | woody |
| Carpinus turczaninowii | woody |
| Carpinus viminea | woody |
| Carpinus viminea var. chiukiangensis | woody |
| Colona floribunda | woody |
| Colona thorelii | woody |
| Cyclocarya paliurus | woody |
| Dalbergia assamica | woody |
| Dalbergia balansae | woody |
| Dalbergia benthamii | woody |
| Dalbergia burmanica | woody |
| Dalbergia candenatensis | woody |
| Dalbergia dyeriana | woody |
| Dalbergia fusca | woody |
| Dalbergia hainanensis | woody |
| Dalbergia hancei | woody |
| Dalbergia henryana | woody |
| Dalbergia hupeana | woody |
| Dalbergia jingxiensis | woody |
| Dalbergia kingiana | woody |
| Dalbergia millettii | woody |
| Dalbergia mimosoides | woody |
| Dalbergia obtusifolia | woody |
| Dalbergia odorifera | woody |
| Dalbergia peishaensis | woody |
| Dalbergia pinnata | woody |
| Dalbergia polyadelpha | woody |
| Dalbergia rimosa | woody |
| Dalbergia sericea | woody |
| Dalbergia stenophylla | woody |
| Dalbergia stipulacea | woody |
| Dalbergia tonkinensis | woody |
| Dalbergia tsoi | woody |
| Dalbergia yunnanensis | woody |
| Dinetus decorus | woody |
| Dinetus dinetoides | woody |
| Dinetus duclouxii | woody |
| Dinetus grandiflorus | woody |
| Dinetus racemosus | woody |
| Dinetus truncatus | woody |
| Dioscorea althaeoides | herbaceous |
| Dioscorea aspersa | herbaceous |
| Dioscorea banzhuana | herbaceous |
| Dioscorea benthamii | herbaceous |
| Dioscorea bicolor | herbaceous |
| Dioscorea biformifolia | herbaceous |
| Dioscorea birmanica | herbaceous |
| Dioscorea bulbifera | herbaceous |
| Dioscorea chingii | herbaceous |
| Dioscorea cirrhosa | herbaceous |
| Dioscorea cirrhosa var. cylindrica | herbaceous |
| Dioscorea collettii | herbaceous |
| Dioscorea collettii var. hypoglauca | herbaceous |
| Dioscorea decipiens | herbaceous |
| Dioscorea decipiens var. glabrescens | herbaceous |
| Dioscorea delavayi | herbaceous |
| Dioscorea deltoidea | herbaceous |
| Dioscorea esquirolii | herbaceous |
| Dioscorea exalata | herbaceous |
| Dioscorea fordii | herbaceous |
| Dioscorea futschauensis | herbaceous |
| Dioscorea garrettii | herbaceous |
| Dioscorea glabra | herbaceous |
| Dioscorea gracillima | herbaceous |
| Dioscorea hemsleyi | herbaceous |
| Dioscorea hispida | herbaceous |
| Dioscorea japonica | herbaceous |
| Dioscorea japonica var. oldhamii | herbaceous |
| Dioscorea japonica var. pilifera | herbaceous |
| Dioscorea kamoonensis | herbaceous |
| Dioscorea linearicordata | herbaceous |
| Dioscorea martini | herbaceous |
| Dioscorea melanophyma | herbaceous |
| Dioscorea menglaensis | herbaceous |
| Dioscorea nipponica | herbaceous |
| Dioscorea nipponica subsp. rosthornii | herbaceous |
| Dioscorea panthaica | herbaceous |
| Dioscorea pentaphylla | herbaceous |
| Dioscorea persimilis | herbaceous |
| Dioscorea persimilis var. pubescens | herbaceous |
| Dioscorea poilanei | herbaceous |
| Dioscorea polystachya | herbaceous |
| Dioscorea scortechinii | herbaceous |
| Dioscorea scortechinii var. parviflora | herbaceous |
| Dioscorea simulans | herbaceous |
| Dioscorea sinoparviflora | herbaceous |
| Dioscorea spongiosa | herbaceous |
| Dioscorea subcalva | herbaceous |
| Dioscorea subcalva var. submollis | herbaceous |
| Dioscorea tentaculigera | herbaceous |
| Dioscorea tenuipes | herbaceous |
| Dioscorea tokoro | herbaceous |
| Dioscorea velutipes | herbaceous |
| Dioscorea wallichii | herbaceous |
| Dioscorea yunnanensis | herbaceous |
| Dioscorea zingiberensis | herbaceous |
| Dipterocarpus gracilis | woody |
| Dipterocarpus retusus | woody |
| Dipteronia dyeriana | woody |
| Dipteronia sinensis | woody |
| Dodonaea viscosa | woody |
| Engelhardtia roxburghiana | woody |
| Engelhardtia serrata | woody |
| Engelhardtia spicata | woody |
| Engelhardtia spicata var. aceriflora | woody |
| Engelhardtia spicata var. colebrookeana | woody |
| Eucommia ulmoides | woody |
| Euonymus acanthocarpus | woody |
| Euonymus acanthoxanthus | woody |
| Euonymus actinocarpus | woody |
| Euonymus aculeatus | woody |
| Euonymus aculeolus | woody |
| Euonymus alatus | woody |
| Euonymus balansae | woody |
| Euonymus bockii | woody |
| Euonymus bullatus | woody |
| Euonymus carnosus | woody |
| Euonymus centidens | woody |
| Euonymus chengii | woody |
| Euonymus chenmoui | woody |
| Euonymus chloranthoides | woody |
| Euonymus chuii | woody |
| Euonymus cornutoides | woody |
| Euonymus dielsianus | woody |
| Euonymus distichus | woody |
| Euonymus echinatus | woody |
| Euonymus euscaphis | woody |
| Euonymus ficoides | woody |
| Euonymus fimbriatus | woody |
| Euonymus fortunei | woody |
| Euonymus frigidus | woody |
| Euonymus gibber | woody |
| Euonymus giraldii | woody |
| Euonymus glaber | woody |
| Euonymus gracillimus | woody |
| Euonymus grandiflorus | woody |
| Euonymus hainanensis | woody |
| Euonymus hamiltonianus | woody |
| Euonymus hukuangensis | woody |
| Euonymus hupehensis | woody |
| Euonymus jinyangensis | woody |
| Euonymus kachinensis | woody |
| Euonymus kengmaensis | woody |
| Euonymus kweichowensis | woody |
| Euonymus laxicymosus | woody |
| Euonymus laxiflorus | woody |
| Euonymus lichiangensis | woody |
| Euonymus lucidus | woody |
| Euonymus lushanensis | woody |
| Euonymus maackii | woody |
| Euonymus macropterus | woody |
| Euonymus microcarpus | woody |
| Euonymus myrianthus | woody |
| Euonymus nanoides | woody |
| Euonymus nanus | woody |
| Euonymus nitidus | woody |
| Euonymus oxyphyllus | woody |
| Euonymus pallidifolius | woody |
| Euonymus parasimilis | woody |
| Euonymus percoriaceus | woody |
| Euonymus phellomanus | woody |
| Euonymus pittosporoides | woody |
| Euonymus potingensis | woody |
| Euonymus prismatomeridoides | woody |
| Euonymus pseudovagans | woody |
| Euonymus rehderianus | woody |
| Euonymus sachalinensis | woody |
| Euonymus salicifolius | woody |
| Euonymus sanguineus | woody |
| Euonymus schensianus | woody |
| Euonymus semenovii | woody |
| Euonymus serratifolius | woody |
| Euonymus spraguei | woody |
| Euonymus szechuanensis | woody |
| Euonymus tashiroi | woody |
| Euonymus tenuiserratus | woody |
| Euonymus ternifolius | woody |
| Euonymus theacolus | woody |
| Euonymus theifolius | woody |
| Euonymus tingens | woody |
| Euonymus tonkinensis | woody |
| Euonymus tsoi | woody |
| Euonymus vaganoides | woody |
| Euonymus vagans | woody |
| Euonymus venosus | woody |
| Euonymus verrucocarpus | woody |
| Euonymus verrucosoides | woody |
| Euonymus verrucosus | woody |
| Euonymus viburnoides | woody |
| Euonymus wilsonii | woody |
| Euonymus wui | woody |
| Euonymus yunnanensis | woody |
| Euptelea pleiosperma | woody |
| Excentrodendron obconicum | woody |
| Excentrodendron tonkinense | woody |
| Firmiana colorata | woody |
| Firmiana danxiaensis | woody |
| Firmiana hainanensis | woody |
| Firmiana kwangsiensis | woody |
| Firmiana major | woody |
| Firmiana pulcherrima | woody |
| Firmiana simplex | woody |
| Fontanesia phillyreoides subsp. fortunei | woody |
| Fraxinus baroniana | woody |
| Fraxinus bungeana | woody |
| Fraxinus chinensis | woody |
| Fraxinus chinensis subsp. rhynchophylla | woody |
| Fraxinus depauperata | woody |
| Fraxinus ferruginea | woody |
| Fraxinus floribunda | woody |
| Fraxinus griffithii | woody |
| Fraxinus hupehensis | woody |
| Fraxinus malacophylla | woody |
| Fraxinus mandshurica | woody |
| Fraxinus odontocalyx | woody |
| Fraxinus paxiana | woody |
| Fraxinus platypoda | woody |
| Fraxinus punctata | woody |
| Fraxinus retusifoliolata | woody |
| Fraxinus sieboldiana | woody |
| Fraxinus sikkimensis | woody |
| Fraxinus sogdiana | woody |
| Fraxinus stylosa | woody |
| Fraxinus trifoliolata | woody |
| Fraxinus xanthoxyloides | woody |
| Getonia floribunda | woody |
| Gonostegia pentandra | woody |
| Gouania leptostachya | woody |
| Gouania leptostachya var. macrocarpa | woody |
| Heritiera parvifolia | woody |
| Hiptage acuminata | woody |
| Hiptage benghalensis | woody |
| Hiptage benghalensis var. tonkinensis | woody |
| Hiptage candicans | woody |
| Hiptage candicans var. harmandiana | woody |
| Hiptage fraxinifolia | woody |
| Hiptage lanceolata | woody |
| Hiptage minor | woody |
| Hiptage multiflora | woody |
| Hiptage tianyangensis | woody |
| Hiptage yunnanensis | woody |
| Hopea chinensis | woody |
| Hopea hainanensis | woody |
| Hopea hongayensis | woody |
| Hopea reticulata | woody |
| Humulus yunnanensis | herbaceous |
| Neuropeltis racemosa | woody |
| Oxyria digyna | herbaceous |
| Oxyria sinensis | herbaceous |
| Parapteropyrum tibeticum | woody |
| Parashorea chinensis | woody |
| Patrinia glabrifolia | herbaceous |
| Patrinia heterophylla | herbaceous |
| Patrinia intermedia | herbaceous |
| Patrinia monandra | herbaceous |
| Patrinia monandra var. formosana | herbaceous |
| Patrinia punctiflora | herbaceous |
| Patrinia rupestris | herbaceous |
| Patrinia scabiosifolia | herbaceous |
| Patrinia scabra | herbaceous |
| Patrinia sibirica | herbaceous |
| Patrinia speciosa | herbaceous |
| Patrinia trifoliata | herbaceous |
| Patrinia villosa | herbaceous |
| Patrinia villosa subsp. punctifolia | herbaceous |
| Plagiopteron chinense | woody |
| Plagiopteron suaveolens | woody |
| Platycarya longzhouensis | woody |
| Platycarya strobilacea | woody |
| Pterocarpus indicus | woody |
| Pterocarya hupehensis | woody |
| Pterocarya macroptera | woody |
| Pterocarya macroptera var. delavayi | woody |
| Pterocarya macroptera var. insignis | woody |
| Pterocarya rhoifolia | woody |
| Pterocarya tonkinensis | woody |
| Pteroceltis tatarinowii | woody |
| Pterocypsela elata | herbaceous |
| Pterocypsela formosana | herbaceous |
| Pterocypsela indica | herbaceous |
| Pterocypsela sonchus | herbaceous |
| Pterocypsela triangulata | herbaceous |
| Pteroxygonum giraldii | herbaceous |
| Ranalisma rostratum | herbaceous |
| Rheum acuminatum | herbaceous |
| Rheum alexandrae | herbaceous |
| Rheum altaicum | herbaceous |
| Rheum australe | herbaceous |
| Rheum compactum | herbaceous |
| Rheum delavayi | herbaceous |
| Rheum forrestii | herbaceous |
| Rheum glabricaule | herbaceous |
| Rheum globulosum | herbaceous |
| Rheum hotaoense | herbaceous |
| Rheum inopinatum | herbaceous |
| Rheum kialense | herbaceous |
| Rheum laciniatum | herbaceous |
| Rheum lhasaense | herbaceous |
| Rheum likiangense | herbaceous |
| Rheum maculatum | herbaceous |
| Rheum moorcroftianum | herbaceous |
| Rheum nanum | herbaceous |
| Rheum nobile | herbaceous |
| Rheum officinale | herbaceous |
| Rheum palmatum | herbaceous |
| Rheum przewalskyi | herbaceous |
| Rheum pumilum | herbaceous |
| Rheum racemiferum | herbaceous |
| Rheum reticulatum | herbaceous |
| Rheum rhabarbarum | herbaceous |
| Rheum rhizostachyum | herbaceous |
| Rheum rhomboideum | herbaceous |
| Rheum spiciforme | herbaceous |
| Rheum subacaule | herbaceous |
| Rheum sublanceolatum | herbaceous |
| Rheum tanguticum | herbaceous |
| Rheum tanguticum var. liupanshanense | herbaceous |
| Rheum tataricum | herbaceous |
| Rheum tibeticum | herbaceous |
| Rheum uninerve | herbaceous |
| Rheum webbianum | herbaceous |
| Rheum wittrockii | herbaceous |
| Rheum yunnanense | herbaceous |
| Rindera tetraspis | herbaceous |
| Rumex amurensis | herbaceous |
| Rumex angulatus | herbaceous |
| Rumex aquaticus | herbaceous |
| Rumex chalepensis | herbaceous |
| Rumex confertus | herbaceous |
| Rumex crispus | herbaceous |
| Rumex dentatus | herbaceous |
| Rumex gmelinii | herbaceous |
| Rumex hastatus | woody |
| Rumex longifolius | herbaceous |
| Rumex maritimus | herbaceous |
| Rumex marschallianus | herbaceous |
| Rumex microcarpus | herbaceous |
| Rumex nepalensis var. remotiflorus | herbaceous |
| Rumex obtusifolius | herbaceous |
| Rumex patientia | herbaceous |
| Rumex popovii | herbaceous |
| Rumex pseudonatronatus | herbaceous |
| Rumex similans | herbaceous |
| Rumex stenophyllus | herbaceous |
| Rumex thyrsiflorus | herbaceous |
| Rumex trisetifer | herbaceous |
| Rumex ucranicus | herbaceous |
| Rumex yungningensis | herbaceous |
| Sagittaria guayanensis | herbaceous |
| Sagittaria guayanensis subsp. lappula | herbaceous |
| Sagittaria lichuanensis | herbaceous |
| Sagittaria natans | herbaceous |
| Sagittaria potamogetifolia | herbaceous |
| Sagittaria tengtsungensis | herbaceous |
| Sagittaria trifolia subsp. leucopetala | herbaceous |
| Securidaca yaoshanensis | woody |
| Shorea assamica | woody |
| Terminalia myriocarpa | woody |
| Terminalia myriocarpa var. hirsuta | woody |
| Thlaspi andersonii | herbaceous |
| Thlaspi cochleariforme | herbaceous |
| Thlaspi perfoliatum | herbaceous |
| Thlaspi yunnanense | herbaceous |
| Tridynamia megalantha | woody |
| Tridynamia sinensis | woody |
| Tridynamia sinensis var. delavayi | woody |
| Tripterygium wilfordii | woody |
| Tristellateia australasiae | woody |
| Ulmus androssowii | woody |
| Ulmus androssowii var. subhirsuta | woody |
| Ulmus bergmanniana | woody |
| Ulmus bergmanniana var. lasiophylla | woody |
| Ulmus castaneifolia | woody |
| Ulmus changii | woody |
| Ulmus changii var. kunmingensis | woody |
| Ulmus chenmoui | woody |
| Ulmus davidiana | woody |
| Ulmus davidiana var. japonica | woody |
| Ulmus elongata | woody |
| Ulmus gaussenii | woody |
| Ulmus glaucescens | woody |
| Ulmus glaucescens var. lasiocarpa | woody |
| Ulmus harbinensis | woody |
| Ulmus laciniata | woody |
| Ulmus lamellosa | woody |
| Ulmus lanceifolia | woody |
| Ulmus macrocarpa | woody |
| Ulmus macrocarpa var. glabra | woody |
| Ulmus microcarpa | woody |
| Ulmus parvifolia | woody |
| Ulmus prunifolia | woody |
| Ulmus pseudopropinqua | woody |
| Ulmus pumila | woody |
| Ulmus szechuanica | woody |
| Ulmus uyematsui | woody |
| Vatica guangxiensis | woody |
| Vatica mangachapoi | woody |
| Zygophyllum fabagoides | herbaceous |
| Zygophyllum iliense | herbaceous |
| Zygophyllum macropterum | herbaceous |
| Zygophyllum oxycarpum | herbaceous |
| Zygophyllum potaninii | herbaceous |
| Zygophyllum pterocarpum | herbaceous |
